# *DICER1* Syndrome: What Do We Know of the Pathogenetic Mechanisms?

**DOI:** 10.3390/cancers17172885

**Published:** 2025-09-02

**Authors:** Floor A. Jansen, Jette Bakhuizen, Lennart Kester, Ronald R. de Krijger

**Affiliations:** 1 Princess Máxima Center for Pediatric Oncology, Heidelberglaan 25, 3584 CS Utrecht, The Netherlands; floor.jansen20@gmail.com (F.A.J.); j.j.bakhuizen-3@prinsesmaximacentrum.nl (J.B.); l.a.kester@prinsesmaximacentrum.nl (L.K.); 2 Department of Genetics, Division of Laboratories, Pharmacy and Biomedical Genetics, Utrecht University, University Medical Center Utrecht, Heidelberglaan 100, 3584 CX Utrecht, The Netherlands; 3 Department of Pathology, University Medical Center Utrecht, Heidelberglaan 100, 3584 CX Utrecht, The Netherlands

**Keywords:** *DICER1*, tumor predisposition syndrome, tissue specificity, pathogenesis, pleuropulmonary blastoma

## Abstract

*DICER1* syndrome is a hereditary cancer predisposition syndrome, characterized by a large range of benign and malignant neoplasms. Patients with *DICER1* syndrome have a broad phenotype, with pleuropulmonary blastoma, Sertoli–Leydig cell tumor, cystic nephroma, cervical embryonal rhabdomyosarcoma, cystic lung lesions, and thyroid follicular nodular disease being the most prevalent manifestations. The syndrome is caused by loss-of-function germline variants in the *DICER1* gene, and *DICER1*-related tumors are characterized by second somatic hotspot variants in the RNase IIIb domain of *DICER1*. *DICER1* encodes an endoribonuclease, which is important for RNA interference. This review describes the molecular mechanism of *DICER1* function and the pathogenetic mechanisms of tumorigenesis. The purpose of this review is to describe the pathogenesis, genotype–phenotype correlation and tissue specificity of *DICER1* syndrome. We conclude that there is a lack of knowledge about the exact molecular mechanisms of *DICER1* function and more research is needed to determine the exact role of this altered protein in relation to pathogenesis.

## Simple Summary

People with a change in the *DICER1* gene have a higher risk of developing rare tumors, yet scientists still do not understand why these tumors only form in certain tissues. This review recapitulates how *DICER1* works in the body and what goes wrong in the process of tumor development. We examine the underlying genetic mechanisms, including how inherited and acquired changes in this gene contribute to cancer. We also look at how the body’s normal gene regulation may fail in specific tissues, leading to uncontrolled cell growth. By comparing *DICER1* syndrome to other inherited cancer conditions, we highlight shared and unique features that may explain this tissue-specific behavior. This research aims to better understand how tumors develop in people with *DICER1* variants and what biological processes are involved. These insights could improve how we study, detect, and potentially treat *DICER1*-related diseases in the future.

## 1. Introduction

*DICER1* syndrome was first described in 2009 when it was associated with a familial form of pleuropulmonary blastoma (PPB) [1]. Pleuropulmonary blastoma is a rare malignancy presenting in childhood. The first clinicopathologic report of PPB was published in 1988 and familial predisposition was recognized in 1996, including the occurrence of neoplasms other than PPB [2,3]. Twenty-five percent of children with this rare lung neoplasm have a family history with other clinical presentations. Associated are medulloblastoma, thyroid neoplasia, malignant germ cell tumors, and others [4].

Through linkage analysis in 2009, Hill et al. discovered that the PPB locus is located on chromosome 14q. *DICER1* was one of the candidate genes associated with this familial lung neoplasm, as it has a known role in lung development [5]. Subsequent sequencing of the *DICER1* gene in families with PPB showed heterozygous *DICER1* germline variants in all families studied. Ten of the eleven families studied harbored a loss-of-function (LOF) germline variant [1]. With this, the *DICER1* syndrome was established and has become the first cancer predisposition syndrome that has a link with a defect in microRNA (miRNA) processing.

The relationship between *DICER1* variants in carriers and the development of multiple neoplastic and non-neoplastic lesions has been well-studied over the past few decades. Researchers quickly realized that *DICER1* variants were not only present in PPB but also in other clinical manifestations of the syndrome [6]. The phenotypic spectrum of *DICER1* syndrome is still the subject of active investigation, and thus it cannot be excluded that additional tumor entities will be discovered to be associated with this syndrome. Since the discovery of the syndrome, a growing list of neoplasms as well as non-neoplastic conditions have been associated with the tumor predisposition syndrome. It affects various organs, often at a young age. The most common tumor manifestations are the following: PPB, Sertoli–Leydig cell tumor (SLCT), cystic nephroma, and cervical embryonal rhabdomyosarcoma (cERMS) [7]. Non-neoplastic conditions that are associated with *DICER1* syndrome are benign cystic lung lesions, thyroid follicular nodular disease, global developmental delay, and macrocephaly, as well as less commonly seen retinal changes and kidney and urinary tract anomalies [8,9,10,11,12]. Table 1 shows the currently known conditions seen in patients with *DICER1* syndrome.

Recent diagnostic techniques such as next-generation sequencing have greatly increased the possibility of diagnosing patients with *DICER1* syndrome. Due to the wider use of next-generation sequencing in pediatric oncology, standard research of *DICER1* is more common. The diagnosis of *DICER1* syndrome is established when a heterozygous germline LOF or suspected *DICER1* LOF variant is found by sequence analysis. This molecular testing approach is sufficient to detect more than 90% of pathogenic variants [15]. In 10% of the cases, the variant cannot be found with sequence analysis, and gene-targeted deletion and duplication analysis are needed as the next steps in the diagnostic process.

*DICER1* syndrome is an autosomal inherited disorder, with incomplete penetrance. Of all patients, about 87% inherited a germline LOF variant from one parent, with 13% of the variants being de novo [15]. The exact prevalence of *DICER1* variants in the population is not well-established, since a lot of pathogenetic variants do not contribute to clinical disease. The prevalence of all *DICER1* pathogenic variants in the normal population was recently studied in 2021, showing a prevalence of people harboring a germline LOF *DICER1* variant, ranging between 1:3700 and 1:4600 [8].

When looking at the epidemiology of the syndrome, a few aspects are essential to highlight. The age of onset is primarily in childhood, although the syndrome can manifest at any age. Examples of two common manifestations of *DICER1* syndrome are PPB, which peaks at around 8 months of age, compared to thyroid follicular nodular disease, which peaks between 10 and 20 years old [16]. For the age ranges of appearance of the various tumors, the reader is referred to Caroleo et al. [16].

The risk of tumorigenesis has been assessed and by age 10, 5.3% of non-proband *DICER1* carriers had developed a neoplasm, whereas by age 50, this percentage is 19.3% [17]. When looking at a possible sex difference, there is an important difference found between females and males, with an elevated risk for specific tumor manifestations seen in females. Female carriers seem to be more frequently affected, because of the higher prevalence of thyroid disease, cervical tumors, and ovarian tumors. Prevalence does not equal penetrance, and the percentage of clinically unaffected patients is relatively high. Females have a 50% lifetime chance of showing no neoplasm formation, while for males this is 80% [14]. Subclinical disease is also not uncommon, since small pulmonary and renal cysts, and tiny thyroid nodules can easily remain undetected. Eighty-seven percent of asymptomatic *DICER1*-mutated patients were shown to have thyroid nodules when an ultrasound was performed, and forty-three percent had lung cysts detected by CT scan [18]. The geographical distribution of *DICER1* syndrome showed no evidence of a higher prevalence in any particular geographic region and the syndrome is not known to affect a specific racial group or ethnicity [19].

Since 2016, minor and major indications for genetic testing and counseling have been stated. Genetic testing is recommended when patients present with one major or two minor indications. Individuals with one minor indication and a family history of major or minor indication are also recommended to undergo genetic testing and counseling. The major and minor indications are listed in Table 1 of “*DICER1 and associated conditions: Identification of At-risk Individuals and Recommended Surveillance Strategies*” and the reader is referred to this table [20].

Surveillance recommendations for *DICER1* patients have been updated in 2021. The Host Genome Working Group of the European branch of the Internation Society of Pediatric Oncology (SIOPE HGWG) and the Clinical Guideline Working Group of the CanGene-CanVar project have collaborated to produce a new surveillance protocol used throughout the whole of Europe [21]. The key recommendations of the surveillance protocol are the following: An annual clinical examination from birth to 20 years of age, with thyroid surveillance, a six-monthly chest X-ray from birth until 8 years of age, a renal ultrasound from birth to 6 years of age, and a thyroid ultrasound every 3 years from 8 to 40 years of age. Added to the surveillance program, the collaboration led to an updated information leaflet, with the aim to raise awareness for possible signs and symptoms of *DICER1*-associated tumor types [21]. The updated guidelines on surveillance for individuals with a germline pathogenic *DICER1* variant were recently published by Schultz et al. in *Clinical Cancer Research*, a journal of the American Association for Cancer Research. The most important difference consisted of earlier initiation of pelvic ultrasounds in girls [22,23]. They advise initiating an every-6-month pelvic ultrasound in girls from birth instead of starting from 8 years of age. Data from the *DICER1* syndrome registries showed that 15% of primary ovarian tumors in individuals with a germline pathogenic variant in *DICER1* were diagnosed prior to 8 years of age. Together, these developments highlight the importance of early genetic testing, continued surveillance, and ongoing research to better understand and manage the diverse and evolving clinical spectrum of *DICER1* syndrome.

## 2. Materials and Methods

A narrative review of the literature was conducted to investigate the molecular mechanisms of *DICER1* syndrome, with particular emphasis on the tissue-specific nature of tumor development. To reflect the thematic scope of the review, a representative PubMed search strategy was formulated, as follows:

(“*DICER1*” [All Fields] OR “*DICER1* syndrome” [All Fields]) AND (“Tumorigenesis” [All Fields] OR “Pathogenesis” [All Fields] OR “miRNA processing” [All Fields]) AND (“Tissue Specificity” [All Fields] OR “Organ Specificity” [Mesh] OR “Gene Expression Regulation” [Mesh])

This search, limited to English-language literature and conducted in April 2025, yielded 181 results. Although this strategy was not used prospectively to define the included references, it accurately reflects the topics covered in this review.

A total of 37 articles were selected based on thematic relevance, particularly regarding *DICER1*-related tumorigenesis, microRNA biogenesis, genotype–phenotype correlation, and hypotheses about tissue specificity. Additional references were identified through manual screening of reference lists and previously known key publications.

Notably, a more specific PubMed query: “Organ specificity” [Mesh] AND “*DICER1* syndrome” [All Fields], returned no results, underscoring the current lack of dedicated research on this specific aspect of *DICER1* syndrome. This further justifies the need for a conceptual review exploring possible mechanisms underlying tissue-specific tumor development in this rare cancer predisposition syndrome.

No formal PRISMA-quality assessment of the studies was conducted, as this review aimed to provide a comprehensive and conceptual overview rather than a systematic synthesis of evidence. Only studies published in peer-reviewed scientific journals were included. The selected articles served as the basis for building a theoretical framework to understand the tissue specificity observed in *DICER1* syndrome.

## 3. *DICER1* Function and Its Role in RNA Interference

### 3.1. Molecular Mechanism

**Figure 1:**
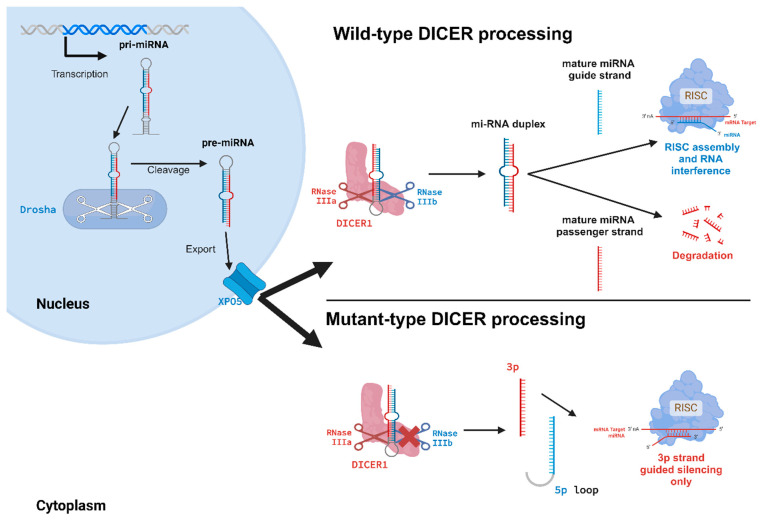
Translation of miRNA genes will lead to the formation of pri-miRNA molecules. Intranuclearly, these pri-miRNAs are processed by Drosha. Drosha, the homolog of Dicer, will cleave pri-miRNAs into pre-miRNAs. These are subsequently exported to the cytoplasm by XPO5. Dicer cleaves pre-miRNAs with its two RNase domains. This will lead to the production of mature miRNA (the 3p-5p duplex). The miRNA duplex is separated into two strands by a helicase. The passenger strand is cleaved and degraded, and the guide strand (usually the 5p) is loaded into the RISC. There it will execute its function by targeting and silencing mRNAs. However, the mutated variant of Dicer will only produce 3p strands. This will give rise to an alternated RNA interference. Created in BioRender. De Krijger, R. (2025) https://BioRender.com/c33g0o5 (accessed on 25 July 2025).

*DICER1* is the gene that encodes for Dicer, an endoribonuclease with a highly conserved function across different species. It exerts multiple functions, most importantly playing an essential role in RNA interference. RNA interference is possible through miRNA biogenesis and the formation of the RNA-induced silencing complex (RISC), as can be seen in Figure 1 [24]. Primary transcripts of miRNAs are transcribed and translated in the nucleus, producing primary miRNAs (pri-miRNAs). These structures are hairpin-folded and cleaved into precursor miRNAs (pre-miRNAs) in the nucleus by Drosha (encoded by *DROSHA*), the nuclear homolog of Dicer. Pre-miRNAs are precursor forms of miRNAs, with a length ranging between 60 and 70 nucleotides. Exportin 5 (XPO5) is responsible for the transportation of pre-miRNAs into the cytoplasm, where Dicer exerts its function. Dicer will bind to the pre-miRNA and will cleave the hairpin-folded RNA at specific spots [25]. This will contribute to the formation of two maturation products: one double-stranded RNA (dsRNA) duplex, which is approximately 20 nucleotides in length, and the hairpin loop. The dsRNA duplex yields a 3′ (3p) and 5′ (5p) mature miRNA strand. The miRNA duplex will bind to Argonaute-2 protein and this will lead to the formation of the RISC. Argonaute-2 will only retain one miRNA strand as the guide strand, the 3p miRNA or 5p miRNA strand [7]. Usually, the 5p miRNA is retained and used in the RISC. Using the guide strand, the RISC will bind with the retained miRNA 5′-end “seed” region to its target mRNA at the 3′ untranslated region (UTR), where the function of the RISC will be executed [26]. The RISC will either contribute to the translational repression of the target mRNA, or it will cause the target mRNA to be degraded. RNA interference is a powerful method to control gene expression in the cell, since one miRNA can regulate multiple mRNAs through imperfect complementary binding [27].

A subset of miRNAs is not required to be processed by Drosha. This subset consists of “miRtrons” and they are embedded in the intronic region of genes. They are produced by an alternative method, which is being processed by the splicing machinery. In contrast, Dicer is needed for the production of all miRNAs, with the only exception being miR-451, its pre-miRNA processed by Ago2 [28].

### 3.2. Functional Domains

**Figure 2:**
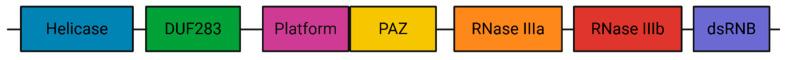
Schematic representation of the most important different domains that build up the Dicer protein. De Krijger, R. (2025) https://BioRender.com/osgy4fl (accessed on 25 July 2025).

**Figure 3:**
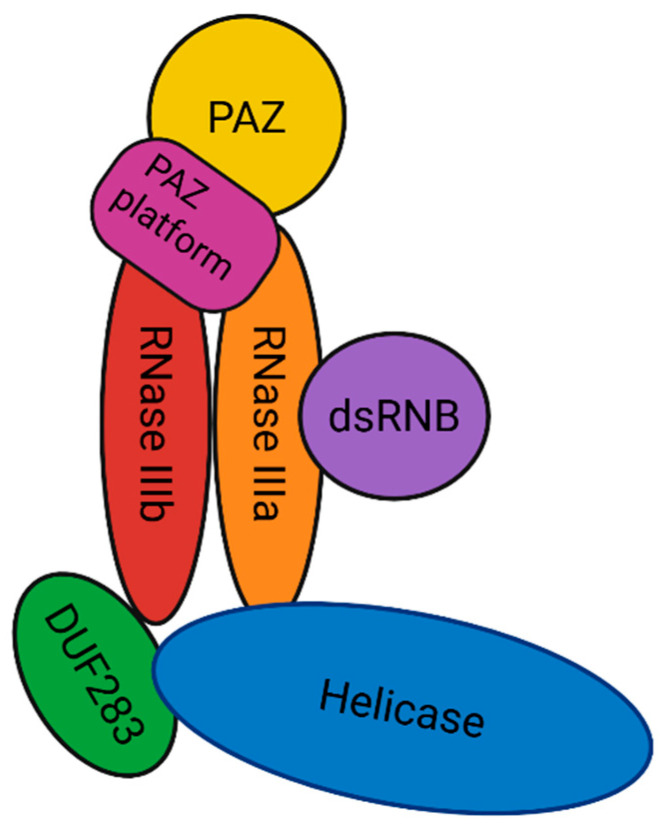
A three-dimensional schematic representation of Dicer. Created in BioRender. De Krijger, R. (2025) https://BioRender.com/zvievwj (accessed on 25 July 2025).

The *DICER1* gene is located on chromosome 14q32.13, is composed of 27 exons, and contains 1922 amino acids. It encodes a well-preserved cytoplasmatic endoribonuclease [29]. Dicer appears to resemble the letter “L”, with a head, body, and base as revealed by cryoelectron microscopy (cryo-EM). Dicer is composed of the following most important domains: A Piwi/Argonaute/Zwille (PAZ) platform domain is located at the head, with a double RNase III domain in the core and a complex helicase domain at the base. Two additional domains are part of the common Dicer architecture: the C-terminal domain responsible for double-stranded RNA-binding (dsRBD) and the DUF283 domain (Figure 2 and Figure 3) [14].

The L-shaped conformation of Dicer results in three major structural regions: the head, the core, and the base. The head consists of the PAZ domain, PAZ/platform domain, and a connecting helicase. The PAZ domain is essential as an RNA-binding domain, as it anchors the overhang of a 2 nucleotide 3′ strand, seen in miRNAs [30]. The PAZ/platform domain is adjacent to the PAZ domain and ensures the combined binding of both strands of the miRNA duplex. The distance between PAZ and RNase IIIa domain is 25 base pairs, so that cleavage occurs at a fixed specific distance from the helical end [29]. This distance is defined by the connecting helix, and it acts as a kind of ruler in the formation of miRNAs.

The core is formed by the dimer of RNase domains. These are essential for the function of Dicer, as they are important for the cleavage function of the protein [31]. The RNase IIIa domain is essential in the cleavage of the 3′ strand of the miRNA duplex, whereas the RNase IIIb domain cleaves the 5′ strand [32]. This will lead to the formation of 3p and 5p miRNAs, respectively. This cleavage is achieved by metal ion-mediated hydrolysis reactions. In the catalytic sites of the RNase domains, Mg^2+^ ions coordinate the hydrolysis reaction with the association of water molecules by transferring protons. The RNase core domains can also be seen in other molecules, such as Drosha. Drosha is also an endoribonuclease but acts earlier in the miRNA biogenesis pathway. It is located in the nucleus and executes the same cleavage function in an earlier part of the miRNA biogenesis pathway [33].

The helicase domain is composed of three subdomains: an N-terminal DExD/H subdomain (HEL1), an insertion domain (HEL2i), and a helicase superfamily C-terminal domain (HEL2). Its main function is substrate recognition and to mediate interaction with other regulatory proteins [34].

The last two domains contain the dsRBD and the DUF238 domain. The function of dsRBD has been established and this domain clamps the double-stranded RNA (dsRNA) in the catalytic sites of Dicer. DUF238′s domain appears to also harbor a dsRBD fold and act similarly to the dsRBD by interacting with the substrate during cleavage [34].

## 4. Genetics

The majority of neoplasms arising in the context of the *DICER1* syndrome, follows an alternative form of Knudson’s “two-hit” hypothesis [29]. *DICER1* is not easily attributed to the tumor suppressor gene or oncogene categories [35]. Tumor suppressor genes are characterized by loss-of-function due to biallelic variants that result in complete inactivation of these genes and the resultant proteins. There are also two mutational events needed to cause the neoplasms associated with *DICER1* syndrome, but there is one important difference to note. In general, one germline LOF variant is found in patients with *DICER1* syndrome, with a second somatic missense variant in the wild-type allele in the RNase IIIb domain [18]. This second variant does not lead to the full inactivation of *DICER1* and therefore it does not classify as a tumor suppressor gene. The variants seen in the cleaving RNase IIIb domain are located in specific “hotspots”. The somatic variant is the rate-limiting step in tumorigenesis as it is a low-probability event, compared to the high-probability event of a LOF variant occurring. Therefore, loss of heterozygosity (LOH) is a rare phenomenon in *DICER1* neoplasms, but has been described in pineoblastomas as explained later on [14]. Somatic variants in *DICER1* typically appear in five specific codons of the endoribonuclease: E1705, D1709, G1809, D1810, and E1813 [29]. These codons are all situated in the RNase IIIb domain and are essential for metal-ion binding, needed for the catalytic cleavage ability. *DICER1* syndrome appears to be a genetically heterogeneous disease, as a broad range of variants can be seen among the germline variants. Missense, nonsense, frameshift, splice-site variants, large duplications or deletions, and even full-gene deletions are all described variants seen in *DICER1* syndrome [36,37]. However, all of these results in loss-of-function of one allele of *DICER1*.

It is important to realize that there are no publications to date describing patients with a germline hotspot variant present in all cells (only cases of mosaicism have been described, as explained later on). This suggests that RNase IIIb domain variants are not viable as germline variants throughout the whole body. It is hypothesized that a fully expressed germline hotspot variant disrupts the embryogenesis process. This is in line with the observation that retention of a portion of *DICER1* activity is needed for tumorigenesis to occur [38]. This is supported by mouse models: a complete knockout of *DICER1* did not lead to tumor formation, but in contrast, led to the inhibition of tumorigenesis [39].

Some exceptions to this model are described in the current literature. One that has been reported is the occurrence of the extremely rare brain tumor pineoblastoma. This tumor has an established relation with *DICER1* syndrome but does not follow the model of tumor formation like other clinical associations. It frequently harbors a second somatic LOF variant, next to the germline LOF variant; therefore, this tumor is characterized by two LOF variants and LOH can be seen in the context of this rare neoplasm [40]. The tumor is devoid of any RNase IIIb hotspot variants. Other exceptions are those of rare cases, where only a single somatic *DICER1* hotspot variant was reported, without LOF variants or allelic loss. These individual cases consisted of Wilms tumors and non-epithelial tumors, and they suggest that a single hotspot variant may be sufficient to promote tumorigenesis [10,41]. It is speculated that the mutated *DICER1* product could have a dominant-negative effect over the wild-type allele [18]. Interestingly, these two tumors (Wilms tumor and pineoblastoma) also harbor *DROSHA* variants, which are also involved in the RNA-silencing pathway [42].

### Mosaicism

One phenotype of *DICER1* syndrome is more aggressive than the other phenotypes described. This has been linked to a specific genotype, namely a mosaic form of RNase IIIb hotspot variant. These patients lack a germline variant throughout the whole body but harbor an RNase IIIb germline variant in a certain percentage of cells. The more cells affected by the variant, the more aggressive the phenotype. These patients differ from other *DICER1* patients in two ways: (1) there is an early presentation of tumor formation and (2) mosaic patients are associated with multifocal disease [43]. Often, multiple neoplastic foci are discovered, involving multiple tissue or organ sites. The explanation of this phenomenon lies in the mutational events needed for the commencement of neoplastic conditions. In patients with a germline LOF variant, a second specific hotspot variant is needed, which is a rare, low-probability event. This event can take months or even years, and the low likelihood of this variant may explain why there is an abundance of clinically unaffected *DICER1* patients. In contrast, the cells affected in mosaic patients already present with the RNase IIIb hotspot variant, and the second mutational event needed is a LOF variant. This second hit is a much higher-probability event, which explains the earlier onset of tumors in this group of patients. Additionally, multiple LOF variants are therefore not unlikely to occur, which explains the multifocality of tumors. When Klein et al. first discovered the mosaic missense variants in *DICER1*, they described a specific phenotype linked to the genotype [10]. The term GLOW syndrome was used, an acronym for Global delay, Lung cysts, Overgrowth, and Wilms tumors. This term is however not accurate and therefore no longer in use to describe this phenomenon, but it is important to note that RNase IIIb mosaicism can lead to overgrowth and developmental delay [43].

Mosaicism with LOF variants has also been described, and this genotype is linked to a very mild form of the disease [43]. Similar to germline LOF variant individuals, the second hit is a low-probability event, and even fewer cells are affected in the mosaic setting. The number of patients with this form of mosaicism is therefore not well established, because it is possible that they do not develop tumors at all in their lifetime.

## 5. Tumorigenesis

It is an attractive hypothesis that other mutational events need to occur, given the low penetrance of the syndrome. Apart from the two mutational events earlier described, it seems that spatiotemporal restriction is found in *DICER1* tumors. Given that the tumors are restricted to specific sites and a specific age of onset is seen in specific tumors, the question arises what other mutational events need to occur to lead to tumor formation. In addition to *DICER1* mutations, other somatic alterations may also contribute to tumorigenesis in these patients. For example, *TP53* mutations have most commonly been reported in PPB, a *DICER1*-associated tumor [44,45]. Other oncogenic alterations have been found, such as *NF1*, *KRAS*, *NRAS*, *FGFR4*, and *EGFR* mutations, suggesting a role of the oncogenic RAS/extracellular signal-regulated kinase (ERK) signaling pathway [46].

However, because *DICER1* syndrome is associated with a broad spectrum of rare tumor types, each occurring in only a small number of patients, recurrent secondary mutations have not yet been identified. As a result, current evidence on the contribution of other somatic variants remains scarce and largely preliminary, given the rarity and heterogeneity of these cancers.

The hotspot variant in the RNase IIIb domain causes Dicer to lose part of its function, whereas 3p miRNA production still occurs because of the intact RNase IIIa domain; the 5p miRNA production is impaired, resulting in an abnormal ratio of 3p and 5p miRNAs [45,47]. Consistent with this finding, it came as no surprise that 5p miRNAs appeared to still be attached to the hairpin loop [48]. This attachment seems to lead to the degradation of 5p strand miRNAs. Independent of the different kinds of variant sites of the hotspot variants involved, it leads to the same shortage of 5p strands [49]. Multiple studies have shown that 5p miRNAs play a more abundant role in the functionality of the RISC, with 3p strands having less contribution to the majority of miRNA activity in humans [50].

**Figure 4:**
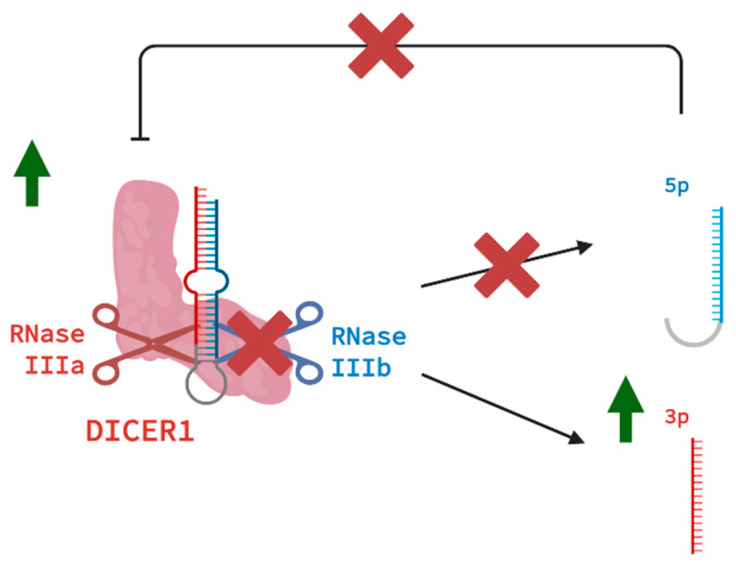
A variant in RNase IIIb leads to an increase in 3p miRNA strands. De Krijger, R. (2025) https://BioRender.com/m69h115 (accessed on 25 July 2025).

To explain why an abundance of 3p strands is seen in *DICER1* neoplasms, the following theory is presented as explained in Figure 4. Several miRNA families are lost due to the deprivation of 5p strand miRNAs in the mutated *DICER1* cells [51]. This includes the Let-7 family, for example [49,52]. Let-7 family members [53], along with miR-9 and miR-146b-5p [54,55], are all downregulated 5p miRNA strands seen in *DICER1* neoplasms. Remarkably, they all target the *DICER1* 3′UTR. Therefore, a negative feedback mechanism targeting *DICER1* is lost when RNase IIIb variants are present. This leads to an increase in mutated *DICER1* activity, which results in an excess of 3p strands [56]. The upregulated set of 3p miRNAs in *DICER1* tumors may be used as a diagnostic tool as they can serve as effective markers in distinguishing thyroid nodules with RNase IIIb variants [49].

### Downstream Effects

It is a challenge to understand completely how variations in *DICER1* gene expression lead to pathogenesis, since its downstream pathways are not completely understood. Loss of miRNAs directly impacts target gene expression downstream and leads to an increase in the expression of these cell signaling pathways. Additionally it is difficult to differentiate genes upregulated through loss of miRNA targeting, or from secondary effects [57]. In general, it has been revealed that impaired miRNA production leads to increased proliferation and enhanced cell migration through collagen matrix in vitro [58]. What specific mechanisms of tumorigenesis occur because of defective miRNA biogenesis? Firstly, it is important to determine which miRNA expression is diminished as a result of *DICER1* variants. As previously reported, the let-7 family is one of the most important miRNA families that are under-expressed in *DICER1* syndrome. MiRNAs are grouped into families, by a shared conserved seed region (essential for target recognition). Along with let-7, miR-26a-5p and miR-125b-5p are also drastically reduced in *DICER1*-mutated SLCTs [56]. Accordingly, a substantially higher expression of target genes of let-7 was seen in *DICER1*-mutated tumors [56,59]. The let-7 family is well-known for its tumor suppressive function, as it downregulates cell proliferation pathways.

Multiple studies revealed that genes upregulated in *DICER1* mutants are associated with cell cycle progression [55,59]. Among the upregulated genes were transcription factors *E2F1* and *E2F5*, various cyclins, and the established marker of cell proliferation, Ki67. To highlight an example, *E2F5* contains predicted binding sites for let-7, mir-17-5p, mir-96-5p, and mir-181-5p, which are all 5p miRNA strands. With the downregulation of these 5p strands, RNA interference cannot take place. *E2F5* expression is increased, which results in prolonged proliferative signaling; a hallmark of cancer [55]. A similar mechanism is seen in the other genes upregulated and associated with cell cycle progression. These were observed in *DICER1* hotspot mutants, with examples such as *CDC25*, *CCNA2*, *CCNE1*, *CDK1*, *SKP2*, and *LIN28A/B* [59].

Specific oncogenes upregulated due to the deprivation of let-7 are *c-Myc* and *K-Ras* [58,60]. The *MYC* proto-oncogene produces the transcription factor MYC, which together with MAX, binds to DNA to regulate gene expression. Activation of *MYC* is correlated with cellular growth and proliferation [61]. *K-RAS* encodes for a GTPase and is a member of the oncogenic Ras family. It has the ability to activate multiple signaling pathways. These can independently act as drivers of tumorigenesis, including PI-3-K, MEK, Ral, mTOR, and p70 S6 kinase [62]. In conclusion, the deprivation of 5p strands due to the mutational events of *DICER1* syndrome leads to increased cell cycle progression and proliferative activity.

Additionally, upregulation of *FN1*, which is negatively correlated with cell differentiation, was found in these tumors. In contrast, *TPO*, *TFF3*, and *LRP2,* which are all positively correlated with differentiation, were downregulated. Therefore *DICER1* also appears to function in the process of cell differentiation [55]. The specific cell signaling pathways, however, have not been well studied yet, so this proves the importance of more research.

Altered RNA silencing is not the only mechanism that has been proposed as a tumorigenic mechanism in PPB. It has been shown that *DICER1*, along with *DROSHA*, plays a role in the repair of double-stranded DNA breaks. In wild-type cells, double-stranded DNA breaks resulted in the relocalization of Dicer to the nucleus via phosphorylation. This phosphorylation is mediated through ERK, as part of the KRAS/MAPK pathway [33]. Upon arrival in the nucleus, Dicer will produce small RNA molecules, aiding in the process of DNA repair. Knockdowns of *DICER1* or *DROSHA* restored DNA replication with the presence of double-stranded DNA breaks. They continued entry into mitosis, and therefore cell cycle progression was restored. RNA products of *DICER1* and *DROSHA* are required for the DNA-damage response pathway to be activated. Knockdown of these two endonucleases also leads to a decreased enforcement of DNA-damage-induced checkpoints. It is still uncertain which domains of *DICER1* and *DROSHA* are responsible for the formation of the small RNA molecules needed for DNA repair. If the RNase IIIa domain is sufficient enough in this production, then this proposed tumorigenic mechanism can be disregarded [63,64].

Taken Together, the Imbalance of 5p miRNAs Remains the Best Supported Mechanism of *DICER1*-Driven Tumorigenesis, While the Proposed DNA Repair Defect Represents an Intriguing but Less Established Pathway, Since It Involves One of the Non-Canonical Functions of *DICER1*. Both May Contribute in Parallel, but Future Research Will Be Required to Clarify Whether This DNA Repair Function Contributes Significantly to *DICER1*-Related Tumorigenesis. 

## 6. The Mechanisms of Tissue Specificity

When looking at *DICER1* syndrome it is remarkable that this tumor predisposition syndrome shows a broad phenotype with common manifestations and clinical conditions. Mendelian diseases frequently show tissue-specific manifestations, although this is not a standard rule. Well-known examples of oncogenic driver variants related to specific tumor sites are seen in multiple hereditary cancer predisposition syndromes [65]. Familial adenomatous polyposis (FAP) is caused by a germline *APC* variant, which results in a nearly 100% lifetime risk of developing colon rectal cancer, in contrast to other tumor types which are relatively rare. Another association is that of patients with *BRCA1* or *BRCA2* gene variants, which cause hereditary breast and ovarian cancer syndrome. Some gene variants lead to a broader spectrum of tumor types, with Li–Fraumeni syndrome being an example of this phenomenon.

The most common assumption is that expressed genes are increased in or exclusive to certain tissues, and variants are the cause of the tissue specificity. Remarkably, this is not always sufficient to explain tumor predisposition syndromes, since diseases caused by heritable traits mostly consist of germline variants [66]. These variants are present throughout the body, yet tumors often arise in a limited number of tissues. This suggests that other factors, such as tissue-specific gene expression, epigenetic context, or cell-type-specific vulnerabilities may contribute to the observed specificity. *DICER1* syndrome is no exception, and the main question arising is what causes the tissue specificity seen in this syndrome.

Some articles proposed molecular mechanisms, which could explain the observed tissue specificity [67,68]. However, the context of tissue specificity seen in tumor predisposition syndromes has only been studied to a limited extent. Together with lessons from other cancer predisposition syndromes, these mechanisms are observed and summarized below. This is followed by the description of a potential correlation between *DICER1* and a serving molecular mechanism.

### 6.1. Lessons from Other Cancer Predisposition Syndromes

Can we learn more from other hereditary cancer syndromes? And can insights from their mechanisms of tissue specificity help us understand *DICER1* syndrome?

The Li–Fraumeni syndrome (LFS), caused by germline *TP53* variants, has a broad phenotype similar to *DICER1*. *TP53*, like *DICER1*, is expressed throughout the body, yet tumors arise in specific tissues. Although the precise mechanism of tissue specificity in LFS remains unclear, various hypotheses have been proposed, including genetic background, environmental influence, and epigenetic regulation. A recent study showed that among LFS patients with germline *TP53* variants, additional germline pathogenic or likely pathogenic variants in other cancer genes were found [69]. This shows the involvement of other genes. In addition, modifier variants in the *Wnt* signaling pathway lead to a decreased cancer incidence. Finally, when looking at the noncoding genome, epimutations in genes including *ASXL1*, *ETV6*, and *LEF1* lead to an increased cancer risk [69]. This still does not explain the broad phenotype seen in LFS, but it adds to the hypothesis that other genetic and epigenetic factors play a role in tumorigenesis.

Similarly, Beckwith–Wiedemann Syndrome (BWS), an epigenetic overgrowth disorder with increased childhood cancer risk, demonstrates that epigenetic mechanisms such as imprinting defects and altered methylation can contribute to tissue-specific tumor development [70,71]. Interestingly, BWS shows a genotype–phenotype correlation where specific genetic variants are linked to certain tumor types. This reinforces the idea that insight into tissue specificity is achievable in some syndromes.

In contrast, the MEN-2 syndrome provides an example of selective gene expression. Activation variants in the RET gene cause tumors specifically in neural crest-derived tissues because RET is naturally expressed there [67]. However, *DICER1* lacks such tissue-restricted expression, making this mechanism unlikely to apply.

Another relevant comparison comes from recent insights into *DROSHA*, a gene involved in the same miRNA-processing pathway as *DICER1.* Remarkably, somatic variants in *DROSHA*, may lead to similar tumor types seen in *DICER1* syndrome. The single missense variant E1147K, which affects the metal binding function of *DROSHA*, has been identified in Wilms tumor, which is also associated with *DICER1* syndrome. This variant leads to the global downregulation of miRNAs seen in this rare pediatric tumor [51]. A novel study also identified a germline pathogenetic variation in *DROSHA*, leading to an autosomal dominant cancer predisposition syndrome [42]. By studying families of patients diagnosed with pineoblastoma and Wilms tumor, they identified *DROSHA* germline variants in the absence of *DICER1* germline variants. A somatic LOF variant combined with a germline LOF variant of *DROSHA*, similarly to the LOH in *DICER1* syndrome, leads to pineoblastoma formation [72,73,74]. It may be hypothesized that variants in these two genes may contribute to a similar pathogenetic cascade leading to tumor formation. At present, however, such mechanistic parallels have only been demonstrated in Wilms tumor and pineoblastoma. Whether *DROSHA* variants play a role in other tumors associated with *DICER1* syndrome remains unknown and requires further research.

### 6.2. Integrating Mechanism of Tissue Specificity

Taken together, these syndromes suggest that tissue specificity in hereditary cancer can arise through different mechanisms.

Expression-based mechanisms, such as exclusive or preferential gene expression, can limit a variant’s phenotypic effects to certain tissues. Additionally post-transcriptional processes can also affect tissue specificity, such as different isoforms of splicing products [75]. While *DICER1* is not exclusively or preferentially expressed in target tissues, expression of mutant proteins or loss of paralogue compensation genes could still play a role. For example, reduced expression of functionally redundant paralogue genes in affected tissues has been found to explain tissue specificity in 43% of 112 cases of tissue-specific hereditary diseases. This mechanisms has been systematically tested on these cases [76].

Regulatory mechanisms, including tissue-specific transcription factor activity and disrupted chromatin organization through altered topologically associated domains (TADs), may also alter gene expression in a tissue specific manner [77]. However, these mechanisms are more common in complex diseases and are less likely drivers in monogenic syndromes like *DICER1* syndrome.

Tissue-disrupted interaction networks likely offer the most plausible explanation in *DICER1* syndrome. Proteins that are broadly expressed, like *DICER1*, may exert different effects depending on their interaction with tissue-specific partners or pathways. For instance, metabolic pathway differences can explain why mitochondrial diseases affect mostly muscles and nerves, and similar pathway-specific vulnerabilities might exist in *DICER1* target tissues.

Despite these hypotheses, there is currently no unified model that explains tissue specificity in *DICER1* syndrome. Multiple studies have shown that there is an absence of tissue specificity for second somatic variants. In addition, it was shown that specific variations in nucleotides or amino acids of the germline LOF variant are not favored in specific tissues affected by the *DICER1* syndrome. Lastly, the type or location of the germline variant has no relation to the position of the second somatic hit [56]. Therefore, it is suspected that local events or simple random events in each tissue play a significant role in comparison to any broader genetic mechanism or systemic pattern since both germline and somatic variants lack tissue specificity [14,78]. However, stochastic events cannot explain tumor formation in its entirety, since *DICER1*-related tumors arise only in a limited set of tissues. This clinical observation suggests that additional, possibly tissue-specific factors may play a role. Nevertheless, current data provide no molecular evidence to support such mechanisms, and their existence remains hypothetical, although their existence cannot be ruled out. The current literature tries to answer the reasons why tissue specificity arises in certain types of cancer [79,80,81], but models for neoplastic disease caused by defective miRNA function simply do not exist.

To date, there has been no proposed molecular mechanism to explain the phenotype seen in *DICER1* syndrome. Since *DICER1* syndrome is a monogenic disease, it is less likely that regulatory mechanisms explain the phenotypical manifestations. In addition, *DICER1* is not preferably expressed in certain tissues [33]. Some authors have speculated that tissue-disrupted networks may play a role, but this remains hypothetical and is not supported by data.

## 7. Conclusions and Future Directions

*DICER1* syndrome is a rare tumor predisposition syndrome, with a prevalence ranging between 1:3700 and 1:4100. The mutational events needed for neoplastic conditions to occur, are part of a “two-hit” model. This consists of a germline LOF variant with a second somatic hotspot variant. Dicer is involved in RNA interference, and the endoribonuclease is essential for the production of miRNAs. The variants seen in *DICER1* syndrome lead to a depletion of 5′ miRNA. This causes downstream effects like continued proliferative signaling, cell cycle progression, and reduced DNA repair. The most important manifestations of this syndrome includes pleuropulmonary blastoma, Sertoli–Leydig cell tumor, cystic nephroma, cervical embryonal rhabdomyosarcoma, and thyroid follicular nodular disease. Since this syndrome is so diverse, treatment is directed at the specific tumor type of the patient. Screening and surveillance aim to find the most prevalent manifestations.

This review aimed to summarize the current literature about a phenotype–genotype correlation in *DICER1* syndrome, in an attempt to answer the question as to why *DICER1* syndrome leads to its specific phenotype, and to find a model for tissue specificity. Because of the rarity of this syndrome, limited data is available from *DICER1* patients. Despite our limited knowledge, certain aspects of tissue specificity in regard to *DICER1* can be noted. Expression-based mechanisms, regulatory mechanisms, and tissue-disrupted networks all lead to tumor specificity. It could be speculated that a disrupted tissue network may lead to the broad range of phenotypes seen in *DICER1* syndrome.

Remarkably, there seems to be a similar tumorigenesis process in *DICER1* syndrome and patients with *DROSHA* variants. *DICER1* syndrome studies are limited and do not explain the exact process of tissue specificity. This is why additional research about *DROSHA* could aid in revealing pathogenetic mechanisms that may also provide insights into tumorigenesis in *DICER1* syndrome. To this day, only Wilms tumors and pineoblastomas seem to arise by the same pathogenetic pathway, but future studies could reveal more associations between the two genes, which are both involved in miRNA biogenesis.

Additionally, it is known that the different manifestations of *DICER1* syndrome have a different age peak. Future research could aim to reveal the temporal part of tissue specificity, since little is known about this phenomenon. Early events that are common among all tumors seen in *DICER1* syndrome could be used as biomarkers and serve in the diagnostic process and prediction of tumor formation. Detection of these biomarkers could come from DNA or RNA extracted from blood samples, since tumors shed cell-free DNA or exomes into the bloodstream. More studies about environmental exposures and their role in tumor formation could aid in creating a better model to predict cancer risk in *DICER1* patients. These environmental contributions are challenging to study because of the limited data available, but they may still play an unknown important role in tumorigenesis in *DICER1* syndrome.

In conclusion, extensive research is still needed to reveal the downstream molecular pathways of *DICER1* involved in tumorigenesis and to create an appropriate model for tissue specificity. This will aid in the general knowledge of *DICER1* syndrome to better predict risk of tumor formation in individual patients and to hopefully find a targeted therapy for these individuals.

## Figures and Tables

**Table 1:** Frequency of *DICER1*-related tumors and tumor-like conditions.

Relative Frequency	Phenotype
Most frequent phenotypes	Type I (cystic) PPB)
	Type II (cystic/solid) PPB
	Type III (solid) PPB
	TFND
	CN
	SLCT of ovary
Moderate frequency phenotypes	DTC
	cERMS
	NCMH
Rare phenotypes	Pituitary blastoma
	Pineoblastoma
	CBME
	Wilms tumor
	Juvenile hamartomatous intestinal polyps
	Bladder ERMS
	ASK
Very rare phenotypes	Mesenchymal hamartoma of the liver
	Medulloblastoma
	Infantile cerebellar embryonal tumor
	oERMS
	Gynandroblastoma
	Cervix primitive neuroectodermal tumor
	Well-differentiated fetal adenocarcinoma

ASK, anaplastic sarcoma of the kidney; CBME, ciliary body medulloepithelioma; cERMS, cervical embryonal rhabdomyosarcoma; CN, cystic nephroma; DTC, differentiated thyroid carcinoma; NCMH, nasal chondromesenchymal hamartoma; oERMS, ERMS of the ovary; OSCST, ovarian sex cord–stromal tumor; PPB, pleuropulmonary blastoma; SLCT, Sertoli–Leydig cell tumor; TFND, thyroid follicular nodular disease. This table is modified from Foulkes et al. and Guillerman et al. [13,14].

## Data Availability

Not applicable.
